# Preclinical studies reveal that LSD1 inhibition results in tumor growth arrest in lung adenocarcinoma independently of driver mutations

**DOI:** 10.1002/1878-0261.12382

**Published:** 2018-10-13

**Authors:** Iris F. Macheleidt, Priya S. Dalvi, So‐Young Lim, Sonja Meemboor, Lydia Meder, Olivia Käsgen, Marion Müller, Karolin Kleemann, Lingyu Wang, Peter Nürnberg, Vanessa Rüsseler, Stephan C. Schäfer, Esther Mahabir, Reinhard Büttner, Margarete Odenthal

**Affiliations:** ^1^ Institute for Pathology University Hospital of Cologne Germany; ^2^ Center for Molecular Medicine University of Cologne Germany; ^3^ Department I of Internal Medicine University Hospital of Cologne Germany; ^4^ Cologne Center for Genomics University of Cologne Germany; ^5^ Lung Cancer Group Cologne University Hospital of Cologne Germany; ^6^ Center for Integrative Oncology University Clinic of Cologne and Bonn Germany; ^7^ Comparative Medicine Center for Molecular Medicine University of Cologne Germany

**Keywords:** epigenetic alterations, HCI‐2509, histone methylation, KDM1A, LSD1, lung adenocarcinoma

## Abstract

Lung adenocarcinoma (LUAD) is the most prevalent subtype of non‐small cell lung cancer. Despite the development of novel targeted and immune therapies, the 5‐year survival rate is still only 21%, indicating the need for more efficient treatment regimens. Lysine‐specific demethylase 1 (LSD1) is an epigenetic eraser that modifies histone 3 methylation status, and is highly overexpressed in LUAD. Using representative human cell culture systems and two autochthonous transgenic mouse models, we investigated inhibition of LSD1 as a novel therapeutic option for treating LUAD. The reversible LSD1 inhibitor HCI‐2509 significantly reduced cell growth with an IC_50_ of 0.3–5 μm *in vitro,* which was linked to an enhancement of histone 3 lysine methylation. Most importantly, growth arrest, as well as inhibition of the invasion capacities, was independent of the underlying driver mutations. Subsequent expression profiling revealed that the cell cycle and replication machinery were prominently affected after LSD1 inhibition. In addition, our data provide evidence that LSD1 blockade significantly interferes with EGFR downstream signaling. Finally, our *in vitro* results were confirmed by preclinical therapeutic approaches, including the use of two autochthonous transgenic LUAD mouse models driven by either EGFR or KRAS mutations. Importantly, LSD1 inhibition resulted in significantly lower tumor formation and a strong reduction in tumor progression, which were independent of the underlying mutational background of the mouse models. Hence, our findings provide substantial evidence indicating that tumor growth of LUAD can be markedly decreased by HCI‐2509 treatment, suggesting its use as a single agent maintenance therapy or combined therapeutical application in novel concerted drug approaches.

**Abbreviations**
EGFR: epidermal growth factor receptor
FAD: flavin adenine dinucleotide
HCI‐2509: N′‐(1‐phenylethylidene)‐benzohydrazide compound
HDAC: histone deacetylase
LSD1: lysine‐specific demethylase 1
LUAD: lung adenocarcinoma
MAO: monoamine oxidases
NSCLC: non‐small‐cell lung cancer
PI: propidium iodide
SCLC: small‐cell lung cancer
TCP: tranylcypromine

## 1 Introduction

Lung cancer is the leading cause of cancer‐related deaths worldwide (Siegel *et al*., 2016). Histologically, lung cancer is divided into small cell lung cancer (SCLC) and non‐small cell lung cancer (NSCLC), which is the most prevalent subtype. Sixty percent of all NSCLC represent lung adenocarcinoma (LUAD). LUAD is characterized by a high frequency of tumor‐driving mutations in gene for epidermal growth factor receptor (EGFR) (10–30%) or KRAS (20–40%) (da Cunha Santos *et al*., 2011). Mutations of the gene for KRAS in codon 12, 13 or 61 result in a constitutive activation of KRAS (Johnson *et al*., 2001) and it is not druggable by clinically applicable ras inhibitors (Pao and Chmielecki, 2010). The two most common mutations detected in the gene for EGFR are a small deletion in exon 19 and a point mutation in exon 21 (L858R), with both activating the receptor tyrosine kinase domain (da Cunha Santos *et al*., 2011).

In addition to the traditional treatment options for LUAD, such as surgery, radiation and chemotherapy, novel approaches, including target‐specific therapy and immunotherapy, have gained more acceptance. However, despite advances in the development of target‐directed therapies interfering with tumor‐driving pathways, the 5‐year survival rate of NSCLC patients still remains at 21% (Siegel *et al*., 2016). Major obstacles with respect to treating NSCLC patients are mainly oncogenic mechanisms (da Cunha Santos *et al*., 2011), which appear in up to 40% of all LUAD cases, comprising of not yet druggable targets such KRAS (Davies *et al*., 2002) or fast occurring resistances against targeted and conventional therapies (Sequist *et al*., 2016). Therefore, further therapeutical strategies are urgently required. Recent reports provide primary evidence of increased chemotherapeutical sensitivity and tumor regression by targeting epigenetic mechanisms such as DNA methylation (Vendetti *et al*., 2015) and histone deacetylation (Fillmore *et al*., 2015; Takashina *et al*., 2016) as summarized previously (Dawson and Kouzarides, 2012; Gelato *et al*., 2016; Tanaka *et al*., 2015). Thus, the alteration of tumor‐associated epigenetic changes is presently considered to be a novel option for NSCLC treatment (Schiffmann *et al*., 2016). One promising option is the inhibition of the lysine‐specific histone demethylase lysine‐specific demethylase 1 (LSD1), also known as KDM1A. LSD1 is an epigenetic eraser that is drastically upregulated in various tumor types (Kahl *et al*., 2006; Schulte *et al*., 2009; Lim *et al*., 2010; Lin *et al*., 2011). In particular, LSD1 overexpression in NSCLC is associated with high malignancy and a poor prognosis (Lv *et al*., 2012; Lim *et al*., 2017).

LSD1 is part of the chromatin remodeling complexes and regulates transcription by specifically targeting and demethylating lysines 4 and 9 of histone 3 (H3K4 and H3K9), which leads to the repression or activation of transcription, respectively (Metzger *et al*., 2005; Lee *et al*., 2006a; Garcia‐Bassets *et al*., 2007). LSD1 consists of three domains: the N‐terminal alpha‐helical SWIRM domain, which functions in chromatin binding, the C‐terminal amine oxidase domain, which harbors the enzymatic activity, and the so‐called tower domain as a protein–protein interaction motif (Zheng *et al*., 2015). The tower domain facilitates LSD1 interaction with coregulators, directing its activity in histone‐modifying multiprotein complexes. Besides its prominent interaction with members of the repressing multiprotein complex, such as the scaffolding corepressor, CoREST and histone deacetylases (HDAC1, HDAC2) (Hwang *et al*., 2011), LSD1 interacts with different transcription factors, such as FOXA1, androgen receptor and STAT6 (Cai *et al*., 2014; Kahl *et al*., 2006; Metzger *et al*., 2005).

Because LSD1 belongs to the family of flavin adenine dinucleotide (FAD) dependent monoamine oxidases (MAO), the demethylation of lysines can be inhibited by MAO inhibitors targeting the FAD‐binding site. Tranylcypromine (TCP) non‐selectively and irreversibly inhibits various MAO and is widely used as an antidepressant (Lee *et al*., 2006b). Although TCP could also inhibit LSD1 at high dosages in breast cancer cells (Lim *et al*., 2010), its clinical application in cancer therapy is limited because of the high concentration needed to inhibit cell growth. Hence, several TCP derivatives with an improved specificity for LSD1 have been developed, such as GSK2879552 and ORY‐1001, which are currently in phase 1 clinical trials for treating acute myeloid leukemia and SCLC (Mohammad *et al*., 2015; Stazi *et al*., 2016).

Using comprehensive screening assays, Sorna *et al*. (2013) identified a *N*′‐(1‐phenylethylidene)‐benzohydrazide compound (HCI‐2509) that selectively inhibits LSD1 with a non‐cellular half maximal inhibitory concentration (IC_50_) of 13 nm. HCI‐2509 not only blocked the FAD‐binding region of LSD1 (Sorna *et al*., 2013), but also abolished LSD1 protein–protein interactions (e.g. with its complex partner CoREST) (Fiskus *et al*., 2014). Furthermore, recent studies demonstrated that treatment with HCI‐2509 efficiently reduced cell growth in prostate cancer, endometrial cancer and Ewing sarcoma (Gupta *et al*., 2016; Sankar *et al*., 2014; Sehrawat *et al*., 2018; Theisen *et al*., 2014).

In the present study, we show that the common MAO inhibitors failed to inhibit cell growth of NSCLC cell types, although HCI‐2509 effectively impedes LSD1 activity, resulting in markedly diminished cell growth and invasion. Most notably, the cell growth inhibition by HCI‐2509 was independent of the tumor‐driving mutations. Thus, it affected the cell growth of different LUAD cell types equally, carrying either an activating KRAS mutation on codon 12, 13 or 61, an EGFR mutation (E746‐A750, L858R, T790M) or an EML4/ALK translocation, in the range 0.3–5 μm in cellular assays. Furthermore, treatment of KRAS or EGFR mutant‐driven transgenic LUAD mouse models revealed that cell growth arrest induced by the HCI‐2509 inhibitor resulted in both lower tumor formation and progression *in vivo*. Using comprehensive expression profiling, we observed that the HCI‐2509‐induced cell cycle arrest is associated with the dysregulation of important factors of cell cycle control and EGFR signaling targets. In conclusion, we propose that HCI‐2509 is a novel drug that could be combined with targeted LUAD therapeutic strategies to prolong the phase of tumor growth arrest, acting independently of the mutation status of the patient.

## 2 Materials and methods

### 2.1 Cell culture and drug treatments

All NSCLC cell lines were a kind gift from Roman Thomas (Department of Translational Genomics, University of Cologne, Cologne, Germany) (Sos *et al*., 2009). A549 and PC9 lung cancer cells were cultured in DMEM (Gibco, Waltham, MA, USA). H1975, H460, H3321 and H2228 lung cancer cells were cultured in RPMI‐1640 (Gibco). All cell culture media were supplemented with 10% fetal bovine serum (Pan Biotech, Aidenbach, Germany).

The LSD1 inhibitors HCI‐2509 (M60160‐2s), RN1 (M60169‐2s), C76 (M66045‐2s), GSK‐LSD1 (M60179‐2s) and OG‐L002 (M60161‐2s) were acquired from Xcessbio (San Diego, CA, USA). Treatment was performed with the inhibitor dosages and duration times indicated, as appropriate. If not otherwise indicated, HCI‐2509 was used at a concentration of 2 μm for 48 h.

### 2.2 Cell growth measurement by MTT and flow cytometric analysis

Cells were plated at a density of 2500 cells per well in 96‐well microplates (Sarstedt, Nümbrecht, Germany). Cell growth was measured by the MTT assay using the Cell Titer 96® Aqueous One Solution Cell Proliferation Assay (Promega, Madison, WI, USA). All tests were performed in technical duplicates and biological triplicates.

Cell cycle arrest was measured by flow cytometry using propidium iodide (PI) staining. Briefly, cells were incubated for 72 h either with or without 2 μm HCI‐2509 in the respective media. Thereafter, cells were trypsinized and collected. After washing with PBS, cells were fixed in ice‐cold 70% ethanol for at least 20 min. The PI staining solution consisted of 20 μg·mL^−1^ PI (Sigma‐Aldrich, St Louis, MO, USA) with 10 μg·mL^−1^ RNase A solution Assay (Promega) and was added 20 min before being measured using the BD FACSCanto™ II (Becton Dickson GmbH, Heidelberg, Germany). The gating strategy is shown in Fig.  S2B.

### 2.3 Invasion assays

For further analysis of the invasion capacities, Boyden chambers (Corning Inc., Corning, NY, USA) were used as described previously (Elfimova *et al*., 2013).

### 2.4 Immunoblotting

Cells were lysed by three freeze‐thaw cycles using cell lysis buffer (New England Biolabs Ipswich, MA, USA) supplemented with 1 mm phenylmethanesulfonyl fluoride (Sigma‐Aldrich). Subsequently, protein concentrations were determined using the Pierce™ BCA Protein Assay Kit (Thermo Fisher, Waltham, MA, USA). Lysates were shock‐frozen in liquid nitrogen and stored at −80 °C until further use. For immunoblotting, using a square slot chamber, 2 μg of protein lysates was added directly onto the nitrocellulose membrane (Bio‐Rad, Hercules, CA, USA) and incubated for 20 min.

Immunoblotting was performed as described previously (Elfimova *et al*., 2013). The antibodies used are shown in Table S1a.

### 2.5 Phosphorylation arrays

To analyze the changes in intracellular signaling after treatment with HCI‐2509, the PathScan EGFR Signaling Antibody Array Kit (#12785; Cell Signaling Technology, Danvers, MA, USA) and the PathScan Intracellular Signaling Phosphorylation Array Kit (#7744; Cell Signaling Technology) were used. First, A549 and PC9 cells were treated for 48 h with 2 μm HCI‐2509 and starved overnight (0.5% fetal bovine serum supplemented DMEM) followed by a stimulation pulse with 100 ng**·**mL^**−1**^ hEGF (Sigma‐Aldrich) for 10 min. Subsequently, cells were lysed using Sandwich Array Lysis buffer (Cell Signaling Technology) supplemented with 1 mm phenylmethanesulfonyl fluoride (Sigma‐Aldrich). Experiments for phosphorylation array analyses were performed in biological triplicates. Phosphorylation signals were read out using the Odyssey^®^ CLx Imaging System from LI‐COR Biosciences (Lincoln, NE, USA).

Alterations observed in response to HCI‐2509 with array technology were validated by western blotting as described above using phosphorylation‐specific antibodies (Table  S1a) and a second set of biological triplicates.

### 2.6 RNA isolation, cDNA synthesis and quantitative PCR

Total RNA from both mouse lungs and cell culture was isolated using the Maxwell® 16 LEV simply RNA Purification Kit (Promega) and quantified using a NanoDrop 1000 spectrophotometer (Thermo Fisher).

cDNA was synthesized using the TaqMan® MicroRNA Reverse Transcription Kit (Life Technologies, Grand Island, NY, USA) in accordance with the manufacturer's instructions.

For real‐time PCR using GoTaq^®^ qPCR Master Mix (Promega), 10 ng of cDNA was used. All reactions were performed in triplicate. Transcripts were normalized to hypoxanthine‐phosphoribosyltransferase (HPRT) transcript levels. Transcripts were calculated by the ΔΔ*C*_t_ method. The primers used for analyses are listed in Table  S2a,b.

### 2.7 Affymetrix microarray procedures

RNA from A549 cells, which were treated for 48 h with 2 μm HCI‐2509 (*n* = 3) or were untreated, were used for expression profiling by means of Affymetrix hybridization microarrays and Hu.Gene 2.0 (Affymetrix, Santa Clara, CA, USA) as described previously (Elfimova *et al*., 2013). Data were subsequently analyzed with expression console software (Affymetrix) using standard RMA settings, and the gene expression profiles were interpreted using transcriptome analysis console software (Affymetrix) with a cut‐off of *P *≤* *0.05 and a fold change of 1.5. The data are available at Gene Expression Omnibus (GEO) (https://www.ncbi.nlm.nih.gov/geo) under http://www.ncbi.nlm.nih.gov/geo/query/acc.cgi?acc=GSE103256.

### 2.8 Preclinical studies of HCI‐2509 using murine LUAD transgenic tumor models

All mouse experiments were conducted in accordance with the approved guidelines of the responsible national authority and the local Governmental Committee for Animal Experimentation (Düsseldorf, Germany; License no: 84‐02.04.2014.A235). All animals were maintained under a 12:12 h light/dark cycle with unrestricted diet and water. Genotyping was performed immediately after weaning by means of PCR analysis. The primer sequences used for PCR genotyping are shown in Table  S3.

C57BL/6N^TG(EGFR L858R) × TG(CC10‐rtTA)^ mice were generated by crossbreeding C57BL/6N^TG(EGFR L858R)^ and C57BL/6N^TG(CC10‐rtTA)^ mice. C57BL/6N^TG(EGFR L858R)^ mice were kindly provided by Katerina Politi (Politi *et al*., 2006) and C57BL/6N^TG(CC10‐rtTA)^ mice (Tichelaar *et al*., 2000) were purchased from Jackson Laboratories (Bar Harbor, ME, USA). For 8 weeks, doxycycline (Alfa Aesar, Haverhill, MA, USA; J63805) was supplied via the drinking water (2 g doxycycline**·**L^**−1**^). Mice were then subjected to X‐ray microcomputed tomography (μCT) and randomly divided into two groups. For an additional 4 weeks, one group (*n* = 7) received control feed, whereas mice from the experimental group (*n* = 8) received the LSD1 inhibitory diet, containing 180 mg HCI‐2509 kg^–1^ feed, ensuring a daily average uptake of 30 mg HCI‐2509 kg^–1^ bodyweight. μCT scans were repeated twice every other week.

The tumor volume was normalized to the mean lung volume of each mouse. The starting value of each mouse was set to 0 and the continued values were calculated accordingly. Mice were sacrificed after 4 weeks of treatment.

The C57BL/6N^(KRAS G12V)^ mice were generated by König *et al*. (2013). Mice aged 8–12 weeks from both sexes were used for the experiments. KRAS mutant expression and tumor growth in the lung were induced by nasal application of AdCre‐expressing adenovirus using 2 **× **10^7^ PFU (Hoelzel Diagnostika, Cologne, Germany) in a total of 40 μL of PBS. Mice were then randomly divided into two groups, receiving either control (*n* = 15) or HCI‐2509 diet (*n* = 16) as described above for 6 weeks. After tumor induction, all animals were monitored daily for signs of discomfort or respiratory distress. After death, the lung, heart, liver, kidney, colon and spleen tissues of control and HCI‐2509‐treated mice were immediately snap‐frozen in liquid nitrogen for cryopreservation followed by DNA, RNA and protein isolation. For histology and immunohistology, tissues were fixed in 4% PBS‐buffered formalin and paraffin‐embedded. All C57BL/6N^(KRAS G12V)^ mice from the experiments were controlled for transgene expression using the KRAS G12V fused luciferase reporter as target. Successful AdCre induction of the C57BL/6N^(KRAS G12V)^ mice was controlled by expression analysis of the KRAS G12V fused luciferase reporter transgene. RNA isolation, including DNase treatment followed by random primed reverse transcription and quantitative PCR (qPCR) targeting the KRAS G12V fused luciferase reporter, was carried out on all lung specimens. Lung biopsies from mice that did not receive adenoviral Cre application were used as a negative control. The relative luciferase expression was normalized by the Δ*C*t method using HPRT as a housekeeping gene. For Δ*C*_t_ calculation of samples, showing no luciferase amplification at all, the luciferase *C*_t_ values were set to 40.

### 2.9 Histochemistry and immunohistochemistry

Hematoxylin and eosin (H&E) staining of lung, heart, liver, kidney, colon and spleen, as well as immunohistochemistry on formalin‐fixed and paraffin‐embedded tissue, was performed as described previously (König *et al*., 2013). The antibodies used for immunohistochemistry are shown in Table  S1b.

## 3 Results

### 3.1 Low efficacy of cell growth inhibition by conventional MAO‐based LSD1 inhibitors

To determine whether LSD1 inhibitors provide a new therapeutic concept for treatment of LUAD, six NSCLC cell lines, which harbor different tumor‐driving genetic alterations such as the common KRAS mutations on codon 12 or 61, the activating EGFR deletion in exon 19 or point mutation in exon 21, as well as two different versions of EML4/ALK translocations, were used for cell growth studies.

First, NSCLC cells were treated for 5 days with conventional MAO‐based LSD1 inhibitors, including the TCP derivatives GSK690, C76, OG‐L002 and RN1. Surprisingly, none of these inhibitors showed efficient growth inhibition, whereas only high doses of RN1 moderately retarded the viability of PC9, A549 and H3122 cells. OG‐L002 affected only PC9 cell growth, whereas GSK690 and C76 did not influence any of the tested cell types (Fig.  S1A–C and Table 1). In agreement, the global methylation status of the LSD1 targets H3K4me2 and H3K9me2 was not enhanced after treatment of PC9 and A549 cells with RN1 and OG‐L002 (Fig. S1D).

**Table 1:** High IC_50_ values in LUAD cell lines after treatment with TCP derivatives^a^

Cell line	Driver alteration	GSK690	C76	OG‐L002	RN1
PC9	EGFR p.ΔE746‐750	∞	∞	30 μm	27 μm
H1975	p.R858L, p.T790M	∞	∞	∞	∞
A549	KRAS p.G12S	∞	∞	∞	7.8 μm
H460	KRAS p.Q61H	∞	∞	∞	1.5 μm
H3122	EML4 E14/ALK E20 (F)	∞	∞	∞	5.1 μm
H2228	EML4 E6/ALK E20 (F)	∞	∞	∞	∞

F, fusion; ∞, > 100 μm.

a Six NSCLC cells lines with different driver mutations were treated with four different TCP derivatives. The half maximal inhibitory concentration (IC_50_) is listed, indicating whether the cell growth could be inhibited or not.

### 3.2 The reversible LSD1 inhibitor HCI‐2509 efficiently inhibits proliferation and invasion of NSCLC cells

**Figure 1:**
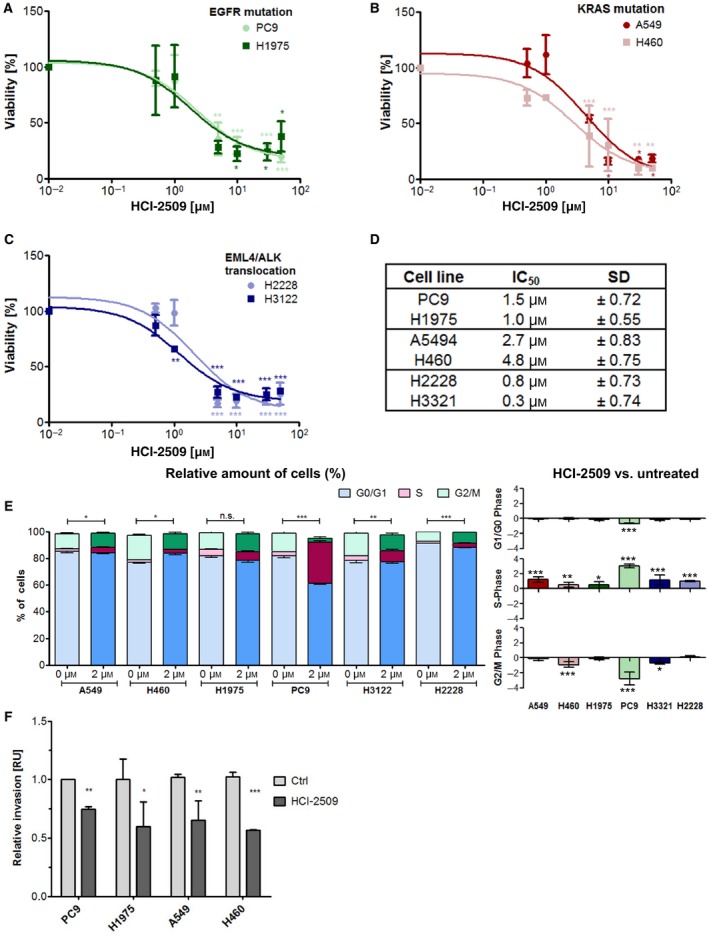
HCI‐2509 reduces the viability and invasion capacities of LUAD cells. (A–D) MTT assay after treating the different cell lines for 5 days with a range of HCI‐2509 concentrations as indicated. The viability of untreated cells (Ctrl) was set to 100%. (A) EGFR‐mutated cell lines PC9 and H1975, (B) KRAS‐mutated cell lines A549 and H460 and (C) cell lines with an EML4/ALK translocation H2228 and H3321. SD and the significances of all values were calculated using analysis of variance (ANOVA) followed by Dunnett's post‐hoc test. (D) Calculated IC_50_ values of NSCLC cells treated with HCI‐2509. (E) Cell cycle analysis by flow cytometry using PI staining of the indicated cell lines (A549, H460, H1975, PC9, H3122 and H2228) treated with 0 or 2 μm HCI‐2509 for 72 h. The percentage of cells (left) was calculated using appropriate gating (Fig.  S2B). The percentage of each cell cycle phase of untreated cells per cell line was set to 0 and the percentage of treated cells was calculated accordingly (right). The significance for the relative amount was calculated by means of two‐way ANOVA (left) and the fold change (right) was calculated using Student′s *t*‐test. (F) Invasion assays performed with Boyden chambers. Invaded cells were stained with crystal violet after 48 h and photographed five times. The pixel value was measured for each photograph and the value of the untreated cells was set to 1. The HCI‐2509‐treated cells were calculated accordingly. Significances were calculated with Student's *t*‐test. Significances are indicated: *P ≤ 0.05, **P ≤ 0.01, ***P ≤ 0.001.

Next, we studied the inhibitory effect of the reversible LSD1 inhibitor HCI‐2509 on cell growth and invasion. By contrast to the MAO‐based LSD1 inhibitors, cell growth of all six cell lines was efficiently reduced in a concentration‐dependent manner by HCI‐2509 (Fig. 1A–C), revealing an IC_50_ of 0.3–5 μm. The time course showed that HCI‐2509 treatment affects cell growth after 48 h (Figs 1D and S2A). Furthermore, cell cycle analysis by PI‐mediated cytometry demonstrated that HCI‐2509 treatment leads to an S‐phase arrest (Figs 1E and S2B). Notably, even at a high concentration, almost no dead cells (up to 5% depending on the cell line) were found (data not shown).

In addition to the profound impact of HCI‐2509 on cell cycle progression, cell invasion was also significantly decreased to approximately 50% (Fig. 1F).

Interestingly, we also observed that HCI‐2509 treatment resulted in a reduction of LSD1 expression after 48 h (Fig.  S2C). In line with that, cycloheximide treatment revealed a shift in the half‐life of LSD1 from 30 h in untreated PC9 cells to 21 h in PC9 cells treated with HCI‐2509 (Fig.  S2D,E).

Because LSD1 inhibition by HCI‐2509 leads to a global change of the H3K4 and H3K9 methylation status (Fig.  S2C), we investigated changes in gene expression profiles underlying cell growth arrest and inhibition of invasion upon LSD1 inhibition.

### 3.3 Inhibition with HCI‐2509 leads to differential expression of key mediators involved in cell cycle and proliferation

To clarify the transcriptional changes after LSD1 inhibition, expression profiles were studied on KRAS‐mutated A549 and EGFR‐mutated PC9 cells treated with 2 μm HCI‐2509 for 48 h. In response to HCI‐2509 treatment of KRAS‐mutated A549 cells, numerous genes (*n* = 890) were differentially expressed, as shown by a comprehensive analysis using hybridization microarrays. In total, 54% of the divergently expressed genes were downregulated (Fig.  S3 and Table  S4).

**Figure 2:**
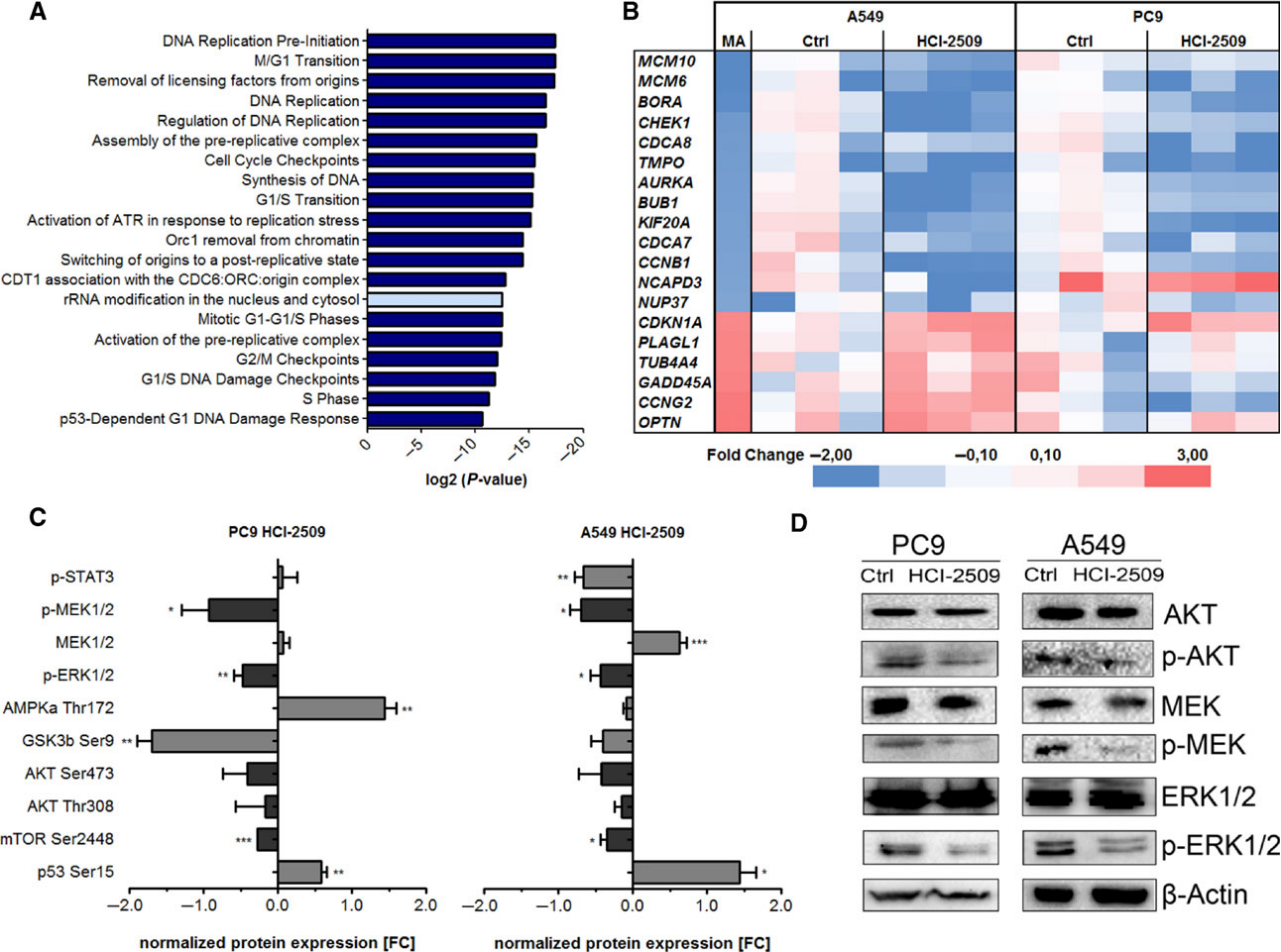
The cell cycle, as well as the EGFR signaling pathway, is dysregulated after HCI‐2509 treatment. (A) The top 20 most significant pathways revealed by reactome pathway analysis. Pathways related to cell cycle control are indicated in dark blue and light blue bars indicate other pathways. (B) Heatmap of expression profiles shown by microarray analysis (MA) were validated by qPCR using RNA of untreated (Ctrl) and HCI‐2509‐treated A549 and PC9 cells. Validation experiments were performed in biological triplicates. (C) Phosphorylation array results in PC9 and A549 cells showing the relative normalized integrated intensity. Values of untreated (Ctrl) cells were set to 0 and HCI‐2509‐treated cells were calculated, respectively. Significances were calculated using Student's *t*‐test. Significances are indicated: *P ≤ 0.05, **P ≤ 0.01, ***P ≤ 0.001. (D) Western blot analysis on PC9 and A549 untreated (Ctrl) and HCI‐2509‐treated (48 h, 2 μm HCI‐2509) immunoblotted against LSD1, AKT, p‐AKT (S373), MEK, p‐MEK, p‐ERK, ERK and the normalization protein β‐actin.

Reactome pathway analysis revealed that differentially expressed genes were involved in cellular replication processes and cell cycle regulation (Fig. 2A). Figure 2A presents the top 20 out of 126 significantly involved pathways and 19 of them are directly or indirectly involved in replication and cell cycle control. Therefore, a total of 20 key factors, which are significantly dysregulated, were selected for further qPCR validation. Indeed, by qPCR, we show that these selected genes, all involved in cell cycle progression and proliferation control, were markedly dysregulated in KRAS‐mutated A549 cells, as well as in the EGFR‐mutated PC9 cells (Fig. 2B).

### 3.4 The LSD1 inhibitor HCI‐2509 impedes EGFR signaling

The EGFR pathway has a big influence on the control of the cell cycle and proliferation. After showing the striking inhibition of cell proliferation by LSD1 inhibition, we further studied the EGFR pathway after HCI‐2509 treatment using a phosphorylation array. A549 and PC9 cells were treated with HCI‐2509 for 48 h and EGFR signaling was then activated by a hEGF stimulation pulse immediately before cell harvest. HCI‐2509‐treated A549 and PC9 cells showed a marked reduction of the phosphorylation status of both Akt proteins, mammalian target of rapamycin, extracellular signal‐regulated kinase (ERK)1/2 and mitogen‐activated protein kinase kinase (MEK)1/2 (Fig. 2C). Especially, the phosphorylated ERK1/2 and MEK forms were strongly reduced, whereas total protein of ERK1/2 and MEK was unchanged upon treatment (Fig. 2C,D).

After demonstrating successfully that inhibition of LSD1 by HCI‐2509 leads to cell cycle arrest and reduction of invasion *in vitro*, we next addressed HCI‐2509 treatment in transgenic murine LUAD models carrying either an EGFR or KRAS mutation.

### 3.5 HCI‐2509 reduces tumor growth in transgenic murine lung adenocarcinoma models

**Figure 3:**
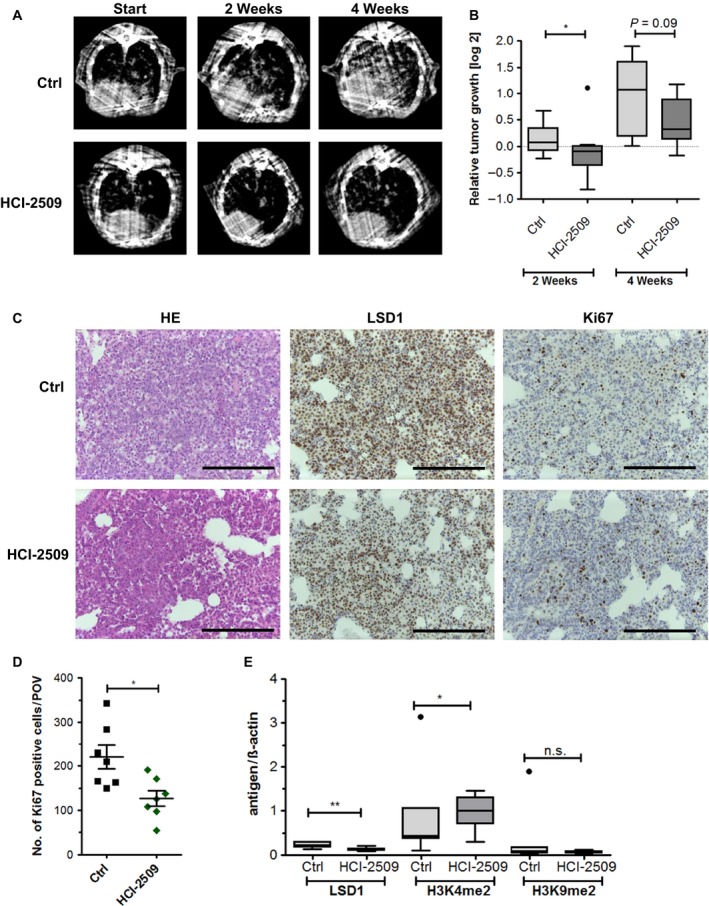
Impeded tumor growth in an EGFR‐driven transgenic mouse model by HCI‐2509 treatment. (A) Exemplary μCT scans of C57BL/6N^TG^ ^(^ ^EGFR^ ^L858R) ×^ ^TG^ ^(^ ^CC^ ^10‐rt^ ^TA^ ^)^ either treated for 2 or 4 weeks with HCI‐2509 or not treated (Ctrl). (B) The relative tumor growths of the control and treated mice were normalized to total lung volume. Significances were calculated using Student's *t*‐test (*P ≤ 0.05). (C) Representative staining (H&E, LSD1 IHC and Ki67 IHC) of EGFR‐driven lung tumors of mice not treated (Ctrl) or with HCI‐2509 treatment over a period of 4 weeks for EGFR‐driven lung tumors. Scale bars = 200 μm. (D) Ki67 expression quantification by counting of total positive cells per view. (E) Immunosquare blot analysis of C57BL/6N^TG^ ^(^ ^EGFR^ ^L858R) ×^ ^TG^ ^(^ ^CC^ ^10‐^ ^RTTA^ ^)^ mice treated for 4 weeks with control feed (*n* = 7) or HCI‐2509 diet (*n* = 8), using antibodies against LSD1, H3K4me2, H3K9me2 and β‐actin. The signals were measured using image lab 4.0.1 (Bio‐Rad) and the signal values of LSD1, H3K4 and H3K9 were normalized using the β‐actin signals. The box plot includes the mean value of each group and each target. Outliers were calculated using Tukey's test and significances were calculated using Student's *t*‐test. Significances are indicated by stars: *P ≤ 0.05, **P ≤ 0.01.

The tet‐on driven transgenic EGFR L858R mutant expression in Clara cells of the C57BL/6N^TG(EGFR L858R) × TG(CC10‐RTTA)^ mice resulted in adenocarcinoma formation throughout the lung. Hence, we induced EGFR L858R expression in C57BL/6N^TG(EGFR L858R) × TG(CC10‐RTTA)^ mice by doxycycline application and, after 8 weeks, we determined the tumor volume. Subsequently, mice were randomly divided into the control and the HCI‐2509‐treatment group, and tumor growth was monitored by μCT every other week (Fig. 3A).

In the control group, strong tumor growth was observed, resulting in duplication of the tumor volume after 4 weeks. By contrast, HCI‐2509 treatment resulted in less tumor progression and we even observed a complete suppression of tumor growth within the first 2 weeks of treatment. However, after 4 weeks, the suppression was less pronounced (Fig. 3B).

As a result of the strong oncogenic driver activity of EGFR L858R expression in all Clara cells, LSD1‐ and Ki67‐positive cells were found throughout the lung of the control mice. In agreement with our μCT studies, upon LSD1 inhibition, the lung tissue was less tightly packed with tumor cells (Fig. 3C). In addition, the number of Ki67‐positive cells was significantly reduced in the lungs of treated mice (Fig. 3D). Corresponding to the slightly but significantly reduced LSD1 levels in lung tissues of HCI‐2509‐treated mice, the H3K4me2 levels were increased, whereas H3K9me2 levels were unchanged (Figs 3E and S4D).

To study the impact of HCI‐2509 on KRAS‐mutated adenocarcinoma *in vivo*, we used C57BL/6N^(KRAS G12V)^ mice, in which the constitutively active KRAS form is induced via adenoviral Cre application. Importantly, this model benefits from sporadic KRAS mutant expression leading to scattered tumor nodule formation and expansion, which resembles the human adenocarcinoma lung cancer type. Mice were divided randomly into control and HCI‐2509‐treated groups upon induction and were weighed regularly throughout the treatment period (Fig.  S4A).

Six weeks postinduction, all C57BL/6N^(KRAS G12V)^ mice were sacrificed and their organs were isolated as described above. All mice were controlled for a positive induction of AdCre and subsequent transgenic KRAS mutant and reporter expression by qPCR (Fig. S4B).

**Figure 4:**
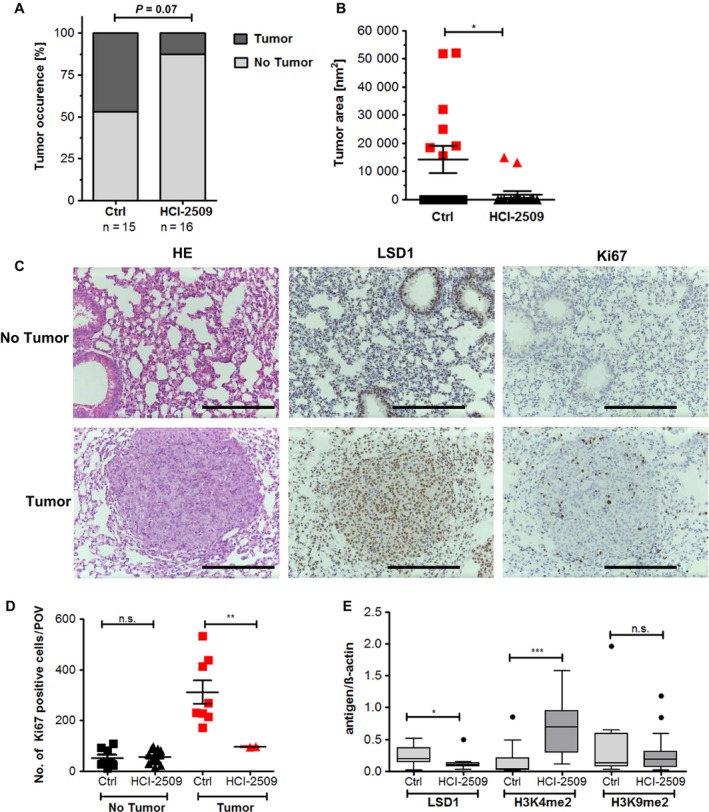
Reduction of tumor growth of KRAS G12V driven LUAD after treatment with HCI‐2509. (A) Tumor occurrence in the control (Ctrl) (*n* = 15) or HCI‐2509‐treated (*n* = 16) group divided by mice showing clear tumor nodules in the H&E‐stained lung half and mice that were tumor‐free. Significances were calculated using Fisher's exact test. (B) The tumor area was measured using cellp software (Olympus, Hamburg, Germany). For mice with tumors, the mean tumor area of all nodules is depicted. Tumor‐free mice have a tumor area of 0. Points indicate single mice, the bars indicate SEM and the significances (*P < 0.05) were calculated by Student's *t*‐test. (C) Example of stainings (H&E, LSD1 IHC and Ki67 IHC) of mouse lungs with tumors and without tumors for two KRAS‐driven control mice. Scale bars = 200 μm. (D) Quantification of Ki67‐positive cells. (E) LSD1, H3K4me2, H3K9me2 protein levels were studied using immunosquare blot analysis of lung tissue lysates of C57BL/6N^(^ ^KRAS^ ^G12V)^ mice treated with control diet (*n* = 15) or HCI‐2509 diet (*n* = 15). The signals were measured using image lab 4.0.1 (Bio‐Rad) and the signal values of LSD1, H3K4 and H3K9 were normalized using the β‐actin signals. The box plot includes the mean value of each group and each target. Outliers were calculated using Tukey's test and significances were calculated using Student's *t*‐test. Significances are indicated by stars: *P ≤ 0.05, **P ≤ 0.01. ***P ≤ 0.001.

Although 47% of the control mice formed tumor nodules, only two out of 16 mice (13%) treated for the entire period with HCI‐2509 developed tumors (Fig. 4A). Additionally, the tumor area was significantly reduced after treatment with HCI‐2509 (Fig. 4B). LSD1 expression was strongly upregulated in areas with tumor nodules compared to pathologically unobtrusive areas (Fig. 4C). In addition, the proliferation marker Ki67 was significantly reduced upon treatment of HCI‐2509. Furthermore, we showed stronger expression of Ki67 in tumor areas than in areas without tumor nodules (Fig. 4D). In agreement with our findings on the LUAD caused by the EGFR tumor‐driving mutation, HCI‐2509 treatment of C57BL/6N^(KRAS G12V)^ mice resulted a significant increase of H3K4me2 levels, whereas H3K9me2 levels were unaffected (Figs 4E and S4E).

## 4 Discussion

In the present study, we demonstrated that the LSD1 inhibitor HCI‐2509 inhibits LUAD cell growth in a dose‐dependent manner *in vitro* and *in vivo*. Because overexpression of LSD1 was observed in many cancer types, including NSCLC, inhibition of LSD1 was suggested as a promising therapeutical option (Lv *et al*., 2012; Lim *et al*., 2017). Surprisingly, in contrast to treatment studies on acute myeloid leukemia and small‐cell lung cancer (Mohammad *et al*., 2015; Stazi *et al*., 2016), conventional MAO‐based LSD1 inhibitors neither affected the histone methylation status, nor influenced NSCLC cell growth. Therefore, we tested the reversible LSD1 inhibitor HCI‐2509, which is a *N*′‐(1‐phenylethylidene)‐benzohydrazide compound. It belongs to the novel class of non‐MAO‐based LSD1 inhibitors (Niwa and Umehara, 2017) acting on LSD1 function in various ways. *In vitro* and *in vivo* studies demonstrate that, in response to HCI‐2509 treatment, gene expression of cell cycle mediators is changed, confirming previous data (Lim *et al*., 2010). Interestingly, this includes the expression of genes that are known to be targets of LSD1 mediated transcriptional regulation, highlighting the direct effects of HCI‐2509. However, there might also be indirect effects of HCI‐2509. Thus, we show that the mitogen‐activated protein kinase pathway is less activated in response to HCI‐2509 treatment. This will, in turn, result in abolishment of cancer‐driven activation of proliferation associated pathways showing the indirect effects of HCI‐2509. Furthermore, HCI‐2509 treatment leads to abrogation of LSD1 protein–protein interactions (e.g. with CoREST) (Fiskus *et al*., 2014). Additionally, we observed that the half‐life of LSD1 is decreased from an initial 30 h to 21 h after HCI‐2509 treatment. This is in agreement with previous studies with respect to neuroblastoma and prostate cancer (Ambrosio *et al*., 2017; Sehrawat *et al*., 2018).

However, unspecific HCI‐2509 function is still a matter of discussion (Mould *et al*., 2015; Sonnemann *et al*., 2017). HCI‐2508 is a derivative of the 4‐hydroxy‐phenyl‐hydrazone core structure that has previously been identified to cause several off‐target hits in several drugs (Baell and Holloway, 2010). Furthermore, the findings of Sonnemann *et al*. (2017) demonstrate a decrease in cell viability in LSD1 knockout cells upon HCI‐2509 treatment, suggesting potential off‐targets of HCI‐2509 that as yet remain unidentified. Nevertheless, in their initial study, Sorna *et al*. (2013) demonstrated the high selectivity of HCI‐2509 towards LSD1.

In our preclinical studies, using NSCLC cell lines, which carry the LUAD‐relevant KRAS and EGFR mutations, HCI‐2509 inhibited cell growth in the IC_50_ range 1–5 μm as measured by cell‐based assays. These confirmational findings were based on prostate and endometrial cancer cells (Gupta *et al*., 2016; Sankar *et al*., 2014; Theisen *et al*., 2014). Furthermore, we show that HCI‐2509 treatment resulted in reduced invasion capacities of NSCLC cell types, although additional effects of the inhibited cell growths could not be excluded because of the experimental set‐up. Importantly, cell growth and invasion was inhibited by HCI‐2509, independently of the activating EGFR or the KRAS mutational status of the NSCLC cell types. In agreement with the high efficiency of HCI‐2509 treatment, regardless of the tumor‐driving EGFR or KRAS mutation, phosphorylation profiling revealed that HCI‐2509 treatment leads to less activation of the EGFR pathway by impeding the phosphorylation of Akt and the KRAS downstream targets, MEK and ERK.

Although HCI‐2509 treatment results in reduced cell growth as a result of cell cycle disruption, we did not observe an increased occurrence of cell death. However, impeded histone demethylation in combination with increased histone acetylation in response to LSD1 inhibition or because of simultaneous treatment with a HDAC inhibitor, leads to the induction of apoptosis in acute myeloid leukemia cells (Zou *et al*., 2017).

Although HCI‐2509 was previously used in cell culture systems and in the corresponding xenograft systems (Gupta *et al*., 2016; Sankar *et al*., 2014; Theisen *et al*., 2014), we applied this reversible LSD1 inhibitor for the first time to genetically engineered mouse models, representing KRAS‐ or EGFR‐mutation‐based LUAD. In the conditional KRAS mutant mouse model, the expression of tumor‐driving mutant depends on viral Cre application, leading to a widespread, but not overall, initiation of tumor nodule formation. In the present study, we demonstrate that HCI‐2509 treatment results in less tumor formation and, importantly, also to less tumor progression. In agreement with the fact that we observed apoptosis neither in cell culture systems, nor in the transgenic LUAD *in vivo* models, no tumor shrinkage was achieved. Hence, LSD1 inhibition by HCI‐2509 might be applied in combined therapeutical strategies of tumor treatment. Indeed, LSD1 inhibition was recently combined with HDAC and EZH2 inhibitors in treatment strategies in acute myeloid leukemia and glioblastoma, as well as in breast and ovarian cancer (Duan *et al*., 2017; Huang *et al*., 2012; Meng *et al*., 2013; Singh *et al*., 2011; Wen *et al*., 2018). However, the treatment approaches in which LSD1 inhibition by HCI‐2509 could be combined with chemotherapeutical agents that induce apoptosis and tumor recession indicate innovative promising concepts. Moreover, HCI‐2509 therapy could be combined with targeted therapies such as treatment approaches with EGFR tyrosine kinase inhibitors. In both scenarios, after tumor shrinkage by chemotherapy or by targeted therapy approaches, HCI‐2509 treatment is assumed to preserve tumor reduction by its function in growth arrest. Thus, repeating chemotherapies with adverse side effects might be reduced and the time frame in which resistance mechanisms develop in response to targeted therapy approaches might be prolonged. Because we did not record any side effects caused by HCI‐2509 treatment, these novel options are suggested to be of extremely high interest.

## 5 Conclusions

In conclusion, our preclinical studies reveal the pharmacological benefits of LSD1 inhibition by HCI‐2509 treatment for novel therapeutical strategies in LUAD as a single agent maintenance therapy or as a combined therapeutical application in novel concerted drug approaches.

## Author contributions

IFM, PSD, RB and MO were responsible for the study conception and design. IFM, PSD, PN and LM were responsible for the development of the study methodology. IFM, PSD, OK, MM, LW, VR, KK, LM, SCS, PN and EM were responsible for the acquisition of data (provided animals, acquired and managed patients, provided facilities, etc.). SCS, IFM and SYL were responsible for the analysis and interpretation of data (e.g. statistical analysis, biostatistics, computational analysis). IFM, SM, EM, RB and MO and were responsible for writing, reviewing and/or revising the manuscript. SM and OK provided administrative, technical or material support (i.e. reporting or organizing data, constructing databases). MO and RB were responsible for study supervision.

## Supporting information


**Fig. S1.** TCP derivatives do not inhibit cell growth of LUAD cell lines.
**Fig. S2.** Treatment of HCI‐2509 results in reduced viability after 48 h and an enhancement of H3K4me2 and H3K9me2.
**Fig. S3.** Treatment of A549 with HCI‐2509 results in dysregulation of the cell cycle by regulating the expression of key regulators.
**Fig. S4.** Adverse side effects and adenoviral Cre application in C57BL/6N(KRAS G12V) mice was controlled.
**Table S1.** (a) List of antibodies used for western blot analysis. (b) List of antibodies used for immunohistochemistry.
**Table S2.** (a) Human primers used for expression analysis by qPCR. (b) Murine primers used for expression analysis by qPCR.
**Table S3.** Primers used for genotyping mouse strains by qPCR.
**Table S4.** Top 100 regulated genes identified using a hybridization micro array after treatment with 2 μm HCI‐2509 in A549 cells.
