# Supplementary material for: Preclinical studies reveal that LSD1 inhibition results in tumor growth arrest in lung adenocarcinoma independently of driver mutations
Source: Mol Oncol. 2018 Oct 13;12(11):1965–79. doi: 10.1002/1878-0261.12382 (PMC6210049; doi:10.1002/1878-0261.12382)
Supplement: Supplementary file 1 — Fig. S1. TCP derivatives do not inhibit cell growth of LUAD cell lines. Fig. S2. Treatment of HCI‐2509 results in reduced viability after 48 h and an enhancement of H3K4me2 and H3K9me2. Fig. S3. Treatment of A549 with HCI‐2509 results in dysregulation of the cell cycle by regulating the expression of key regulators. Fig. S4. Adverse side effects and adenoviral Cre application in C57BL/6N(KRAS G12V) mice was controlled. Table S1. (a) List of antibodies used for western blot analysis. (b) List of antibodies used for immunohistochemistry. Table S2. (a) Human primers used for expression analysis by qPCR. (b) Murine primers used for expression analysis by qPCR. Table S3. Primers used for genotyping mouse strains by qPCR. Table S4. Top 100 regulated genes identified using a hybridization micro array after treatment with 2 μm HCI‐2509 in A549 cells. [file MOL2-12-1965-s001.pdf]

SUPPLEMENTAL INFORMATION

Preclinical studies reveal that LSD1 inhibition results in tumor growth arrest in lung adenocarcinoma independently of driver mutations

Iris F. Macheleidt[1,2], Priya S. Dalvi[1,2], So-Young Lim[1,2], Sonja Meemboor[1,2], Lydia Meder[2,6], Olivia Käsgen[1,2], Marion Müller[1], Karolin Kleemann[1,2], Lingyu Wang[1,2], Peter Nürnberg[5], Vanessa Rüsseler[1,4], Stephan C. Schäfer[1,3,4], Esther Mahabir[7], Reinhard Büttner[1,2,3,4], and Margarete Odenthal[1,2,4]

[1]Institute for Pathology, University Hospital of Cologne, Germany, [2]Center for Molecular Medicine, University of Cologne, Germany, [3]Center for Integrative Oncology, University Clinic of Cologne and Bonn, Germany, [4]Lung Cancer Group Cologne, University Hospital of Cologne, Germany, [5]Cologne Center for Genomics, University of Cologne, Germany, [6]Department I of Internal Medicine, University Hospital of Cologne, [7]Comparative Medicine, Center for Molecular Medicine, University of Cologne,, Germany

**Supplemental Tables**	Supplemental Table S1a, S1b
	Supplemental Table S2a, S2b
	Supplemental Table S3
	Supplemental Table S4
**Supplemental Figures**	Supplemental Figure S1
	Supplemental Figure S2
	Supplemental Figure S3
	Supplemental Figure S4

Supplemental Tables

Supplemental Table S1a: List of Antibodies used for Western Blot analysis

Antibody	Entity	Dilution	Number and Manufacturer
ERK1/2	rabbit	1:3000	M 5670, Sigma-Aldrich, Taufkirchen, GER
H3K4me2	rabbit	1:1000	ab32356, Abcam, Cambridge, UK
H3K9me2	mouse	1:1000	ab1220, Abcam, Cambridge, UK
LSD1	rabbit	1:1000	ab37165, Abcam, Cambridge, UK
p-AKT	rabbit	1:1000	#9271, Cell Signaling, Frankfurt, GER
AKT	rabbit	1:1000	#4691, Cell Signaling, Frankfurt, GER
p-ERK1/2 S	rabbit	1:1000	#4376, Cell Signaling, Frankfurt, GER
p-MEK	rabbit	1:1000	#2338, Cell Signaling, Frankfurt, GER
MEK1/2	rabbit	1:500	#9122, Cell Signaling, Frankfurt, GER
β-Actin	mouse	1:5000	A4551, Sigma-Aldrich, Taufkirchen, GER
α-mouse, HRP	rabbit	1:2000	ab6728, Abcam, Cambridge, UK
α-rabbit, HRP	goat	1:2000	ab6721, Abcam, Cambridge, UK

Supplemental Table S1b: List of Antibodies used for immunohistochemistry

Antibody	Entity	Dilution	Number and Manufacturer
Ki67	rabbit	1:500	ab15580 Abcam, Cambridge, UK
LSD1	rabbit	1:500	ab37165 Abcam, Cambridge, UK
α-rabbit	goat	1:100	Histofine® Simple StainTM Mouse MAX PO (R), Nichirei Bioscience Inc., Tokyo, Japan

Supplemental Table S2a: Human primers used for expression analysis by qPCR

Oligonucleotide	Sequence
18S-F	AAA CGG CTA CCA CAT CCA AG
18S-R	CCT CCA ATG GAT CCT CGT TA
AURKA-F	CAG GCA ACC AGT GTA CCT CA
AURKA-R	GCC AGT TCC TCC TCA GGA TT
BORA-F	CCT TGT GAA AGC AGT AAC ATT CA
BORA-R	TGC ATC ACT TTC TTT GCA GTA AC
BUB1-F	CGG CTT CTA GTT TGC GGT TC
BUB1-R	TAG CTC TGC ATG TGG GCT TC
CCNB1-F	TTT CGC CTG AGC CTA TTT TG
CCNB1-R	GCA CAT CCA GAT GTT TCC ATT
CCNG1-F	TGG ACA GGT TCT TGG CTC TT
CCNG1-R	GAT GGA ATA TTG CAG TCT TCT TCA
CDCA7-F	TCG TTC CGT GGA AGA CAT C
CDCA7-R	ACG AGC TCT CCG TTC AGG
CDCA8-F	ACG TAA GAA TCT TCA AAC TGC AAG
CDCA8-R	CCT TTT CCT TGT ATG GAC TGA GTT
CHEK1-F	CAG GAG AGA AGG CAA TAT CCA A
CHEK1-R	GCA TGC CTA TGT CTG GCT CT

GADD45A-F	GAG AGC AGA AGA CCG AAA GC
GADD45A-R	AGC CAC ATC TCT GTC GTC GT
HPRT-F	GAC CAG TCA ACA GGG GAC AT
HPRT-R	GTG TCA ATT ATA TCT TCC ACA ATC AAG
KIF20A-F	TCA GAG GTC AAG CAG AAG CG
KIF20A-R	AGC TAC CAA TCC GCG TAA GG
LSD1-F	CCC TTA AGC ACT GGG ATC AG
LSD1-R	ACA CGA GTA GCC ATT CCT TAC TG
MCM10-F	TCG AAG TTT CCT GTC ACA ACT G
MCM10-R	GCG GTC AGC AGA GAC AGA TT
MCM6-F	GGG ACC TTT CTT ATA GGC TGG T
MCM6-R	TCC TCA TCT CTG AGC TCT TTC C
MDM2-F	GAG CTT GGC TGC TTC TGG
MDM2-R	GAA AGC AGC AGG ATC TCG GT
NCAPD3-F	CGA GCT TTG AAA GTT GAG CAC
NCAPD3-R	ACA CTG TGT CAA CCC ATT CG
NUP37-F	GTG CAA ACA GCT CAT TTT GTT C
NUP37-R	TCC ATT CTT CTC TGC AAC CAT
OPTN-F	CGG CTC CTC AGA AGA TTC CT
OPTN-R	GAT TTC TTT TAC TGA CCC TTC TGC
CDKNA1-F	CCG AAG TCA GTT CCT TGT GG
CDKNA1-R	CAT GGG TTC TGA CGG ACA T
PLAGL1-F	CAG GGA TTG CTG TCA CGT C
PLAGL1-R	GCA ACG AAA AGT CTT GTC TAA TCA
TMPO-F	CGG ACT TCT CCA GTG ACG A
TMPO-R	TTC TTG TCT GGG TTT ATC AGT TTT T
TUBA4A-F	ACC TGT CAC CCC GAC TCA
TUBA4A-R	ATT GCC CAT CTG GAC ACC T

Supplemental Table S2b: Murine primers used for expression analysis by qPCR

Oligonucleotide	Sequence
HPRT-F	CCTCCTCAGACCGCTTTTT
HPRT-R	AACCTGGTTCATCATCGCTAA
LSD1-F	TGAGCAGATTGAACATTGGAA
LSD1-R	TTGCTGATGGAGCTCTTTAATTT

Supplemental Table S3: Primers used for genotyping mouse strains by qPCR

PCR-Type	Name and target	Sequence
	CRP forward	ACC CAC ATT GAT TTC TCT GTT CTA
	CRP reverse	AAT GAT TTC CTA ACA CTG CCT CTT
**qPCR**	Luc forward	CGA TGA CGG AAA AAG AGA TCG TG
	Luc reverse	GAC CTT TCG GTA CTT CGT CCAC
	CC10 Promoter forward	CCA GCC TCT GGT TCT CCA GGG
	CC10 Promoter reverse	GTC GGG GAA CCC AGA GGT CG
**qPCR**	tetO forward	GTC GAG TTT ACC ACT
	tetO reverse	GAG TGG TAA ACT CGA

Supplemental Table S4: Top 100 regulated genes identified using a hybridization micro array after treatment with 2 μM HCI-2509 in A549 cells

Gene Symbol	Fold Change	ANOVA p-value	FDR p-value
HIST1H2BM	-3.63	0.002084	0.107932
HIGD1C	-3.39	0.010598	0.201773
ST8SIA4	-2.94	0.001032	0.088419
PLA2G4A	-2.86	0.000371	0.070141
CPS1-IT1	-2.83	0.010417	0.200694
LOC105378728	-2.83	0.000158	0.053762
METTL15	-2.73	0.002461	0.115497
LOC101927139	-2.65	0.000792	0.085135
GINS2	-2.63	0.004154	0.144105
KLHL5	-2.63	0.013688	0.220189
FBXW10	-2.56	0.00148	0.098683
HIST1H1D	-2.56	0.000094	0.053762
NTRK3	-2.56	0.000402	0.071859
SNAR-E	-2.55	0.001017	0.088419
ANXA8L1	-2.52	0.016122	0.234196
HIF1A-AS1	-2.51	0.000286	0.06542
HELLPAR	-2.50	0.026211	0.283809
LIN28B	-2.50	0.012109	0.212989
SNORD18A	-2.48	0.004235	0.144861
DDX46	-2.44	0.047219	0.351956
SNORD14E	-2.42	0.003145	0.128037
CKMT1A	-2.39	0.005083	0.153472
ASB4	-2.38	0.001016	0.088419
GRB14	-2.38	0.000186	0.057419
SNORA38B	-2.31	0.027962	0.289748
HIST1H2AI	-2.29	0.014254	0.224208
HIST2H2AB	-2.29	0.018302	0.248064
PATE3	-2.29	0.037734	0.321943
SNORD12C	-2.29	0.019876	0.254812
LOC105376081	-2.26	0.011605	0.209459
PET100	-2.26	0.010399	0.20067
TTC21B	-2.26	0.00335	0.132392
HIST1H2AM	-2.25	0.02954	0.295237
MCM10	-2.24	0.000573	0.078962
SNORD14A	-2.23	0.033243	0.307065
SNAR-D	-2.21	0.004525	0.149361
MCM6	-2.20	0.000152	0.053762

SNAR-B2	-2.20	0.00181	0.105909
RIOK3	2.19	0.000168	0.054864
CREG2	2.21	0.00186	0.105909
TMEM47	2.21	0.001108	0.089487
PAM	2.22	0.000015	0.039233
TMEM156	2.22	0.000556	0.078355
ZNF841	2.23	0.001752	0.105749
CLEC2B	2.24	0.001071	0.089239
RNVU1-3	2.26	0.01972	0.254616
LOC105374715	2.28	0.028624	0.292027
SERPINE2	2.29	0.000015	0.039233
TMEM92-AS1	2.31	0.008009	0.178725
MDM2	2.32	0.000093	0.053762
SLC2A1	2.32	0.000805	0.085135
SNORD114-16	2.33	0.005453	0.156492
LOC151760	2.34	0.000359	0.06922
INSIG2	2.35	0.001506	0.098683
VLDLR	2.35	0.000214	0.059851
THSD1	2.36	0.000856	0.085135
ABCA12	2.37	0.000124	0.053762
APOBEC4	2.37	0.01045	0.200787
LOC105369301	2.38	0.01079	0.203432
WDR63	2.39	0.018191	0.247186
SLFN5	2.40	0.000015	0.039233
NEFL	2.41	0.011816	0.210876
SDC4	2.41	0.001907	0.105909
SNORD114-17	2.44	0.00777	0.176756
MOXD1	2.45	0.000153	0.053762
LOC105370924	2.56	0.001047	0.088546
RRAGD	2.69	0.001708	0.10416
LOC105375451	2.71	0.031399	0.299911
ITGB3	2.73	0.000008	0.036621
TMEM255A	2.73	0.000476	0.076703
PBOV1	2.75	0.001911	0.105909
IGFBP1	2.76	0.004407	0.147518
SNORD114-6	2.80	0.036139	0.317165
SLAMF7	2.85	0.001751	0.105749
ANKRD1	2.88	0.002531	0.116599
TMEM45A	2.88	0.000228	0.061397
PDK1	2.92	0.000272	0.064
SNORD114-12	2.92	0.030164	0.296396
PLIN2	2.93	0.000124	0.053762

P4HA1	2.96	0.000008	0.036621
TGFBR1	2.96	0.000004	0.036621
SPATA18	2.97	0.000131	0.053762
BNIP3	3.04	0.000093	0.053762
TP53INP1	3.08	0.000018	0.039461
PPP1R3C	3.15	0.001369	0.09668
ROS1	3.18	0.00011	0.053762
NDRG1	3.27	0.00093	0.086571
STC2	3.45	0.002928	0.123223
ENTPD4	3.50	0.019209	0.251869
SNORD114-30	3.51	0.044824	0.343885
LOC541472	3.54	0.006011	0.160347
SNORD114-7	3.71	0.031051	0.298793
SNORD114-2	3.75	0.019701	0.254616
CP	3.90	0.000259	0.0633
HIST1H4H	4.04	0.000247	0.062577
IGFBP3	4.16	0.009734	0.195396
SERPINE1	4.35	0.000362	0.06922
SLC2A3	4.45	0.000387	0.070695
STC1	4.48	0.000112	0.053762
LOC101927501	4.80	0.000616	0.08139

Supplemental Figures

Supplemental Fig. S1

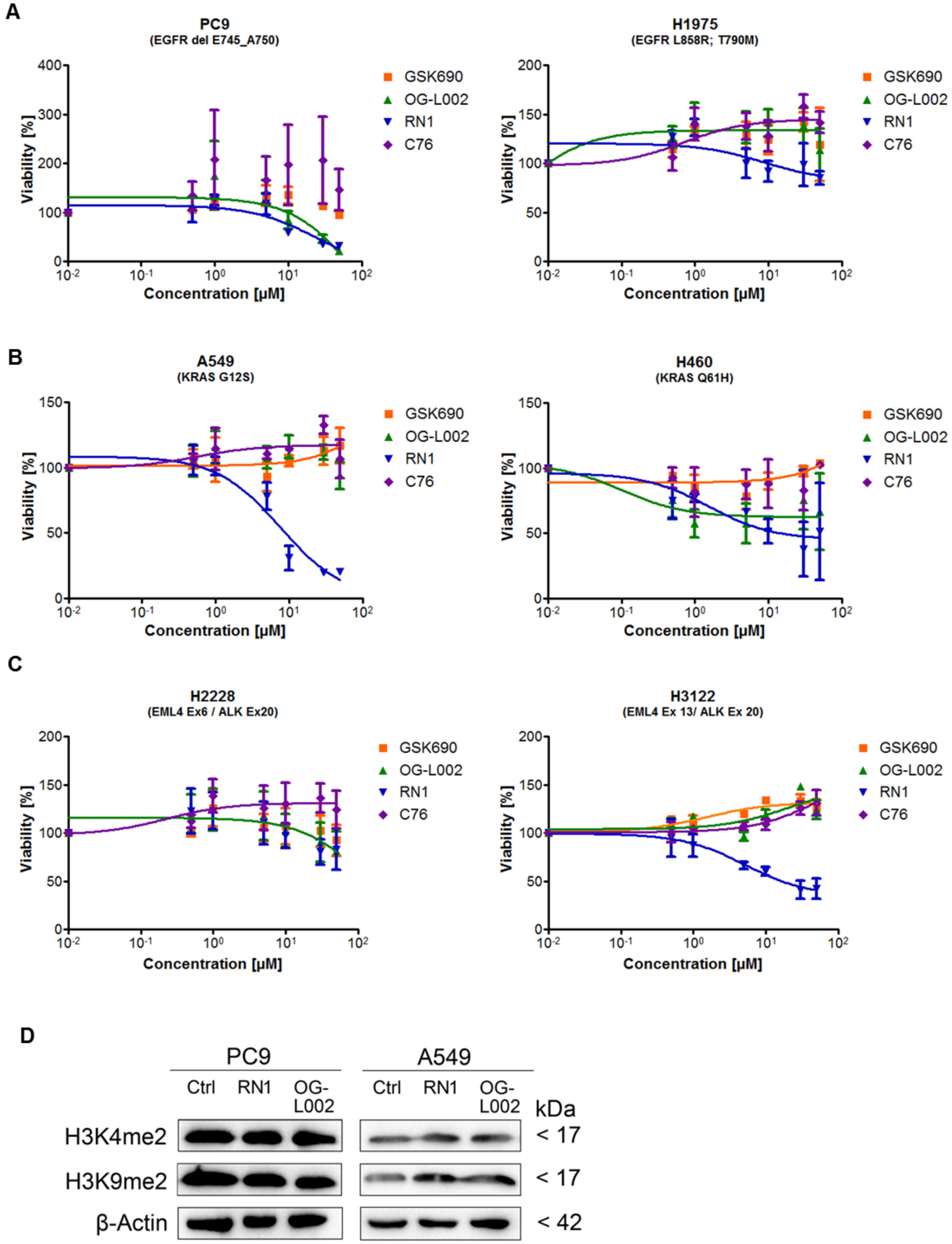

Supplemental Figure S1: TCP derivatives do not inhibit cell growth of LUAD cell lines. MTT assays after treating different NSCLC cell lines for 5 days with concentrations ranging between 0.5 µM and 50 µM of GSK690, OG-L002, RN1 and C76. The viability of untreated cells (= control) was set to 100%. (A) EGFR-mutated cell lines PC9 and H1975 (B) KRAS-mutated cell lines A549 and H460 (C) cell lines with an EML4/ALK translocation H3321 and H2228. (D) Western Blot analysis of PC9 and A549 cells, either untreated (Ctrl) or treated with RN1 (2.5 µM) or OG-L002 (12.5 µM) for 24 h against H3K4me2 and H3K9me2. The protein content was normalized to the expression of β-actin.

Supplemental Fig. S2

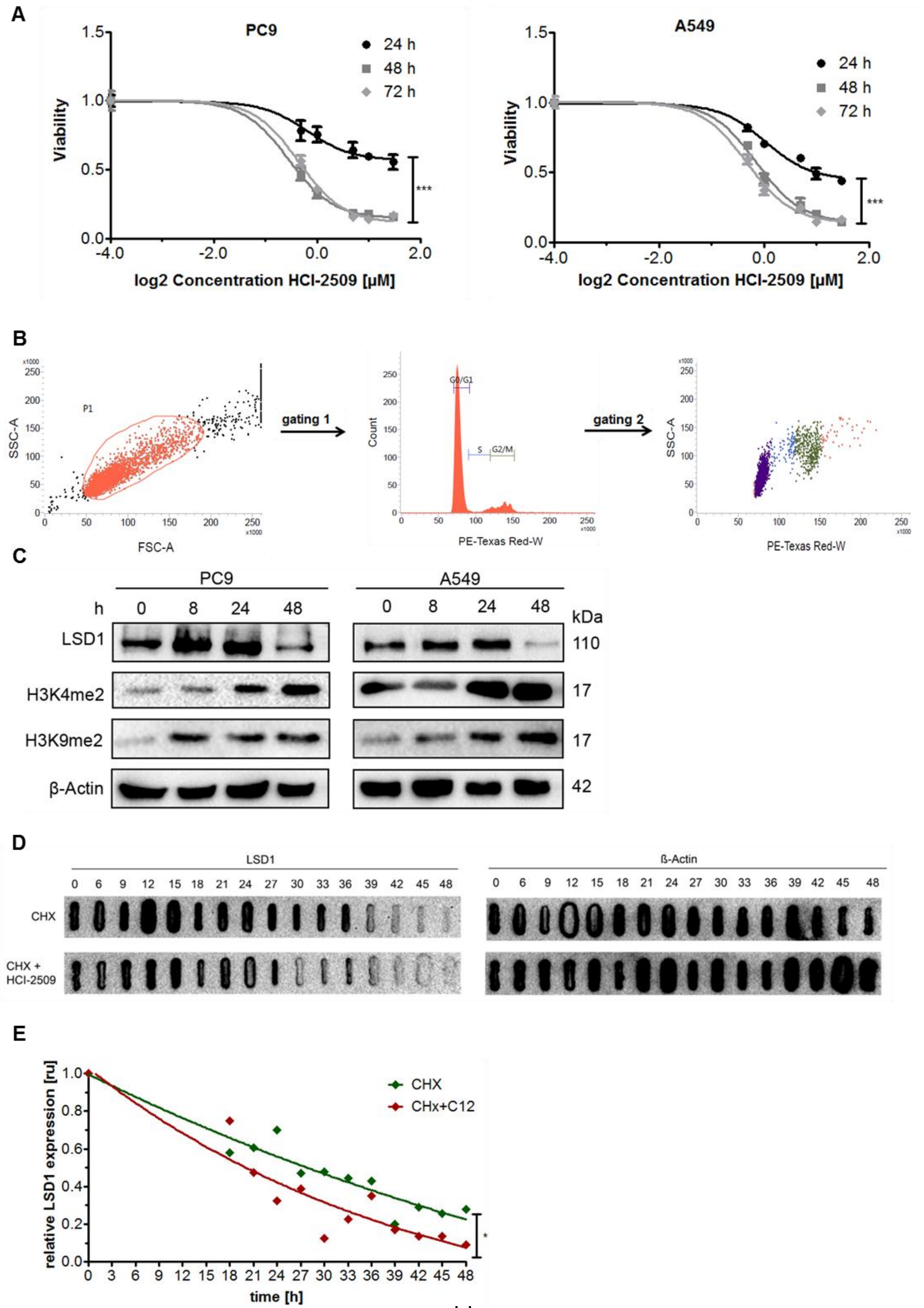

Supplemental Figure S2: Treatment of HCI-2509 results in reduced viability after 48 h and an enhancement of H3K4me2 and H3K9me2

(A) MTT assay after treating the different cell lines for 24, 48 and 72 h with a range of HCI-2509 concentrations as indicated. The viability of untreated cells was set to 1.

(B) Gating strategy for propidium iodide mediated cytometry. Viable single cells were selected by gating 1, whereas the cells in the specific cell cycle phases were selected by gating of the resulting histogram (gating 2). The data of the percentage of cells per cycle was extracted used for further analysis.(C) Western Blotting of PC9 and A549 cells after treatment of 2 µM HCI-2509 for 0, 8, 24 and 48 hours using antibodies against LSD1, H3K4me2, and H3K9me2. Protein contents were normalized to β-actin (D) Immuno-square blotting of PC9 cells treated with either 50 µg/ml cycloheximide (CHX) or CHX in combination with 2 µM HCI-2509 for the indicated time points. Immuno-square blotting was performed using LSD1 and ß-actin antibodies. (E) The signals were measured using Bio-Rad Image Lab 4.0.1. The target signals were divided by the ß-actin signals for each sample. The ratio for the untreated control (0 h) was set to 1 and the other time points were calculated accordingly.

Supplemental Fig. S3

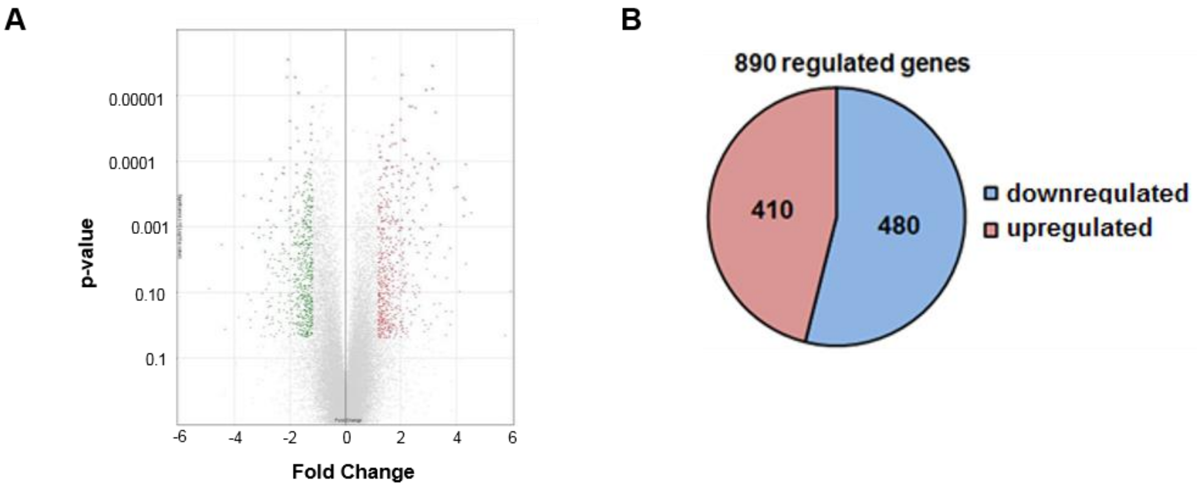

Supplemental Figure S3: Treatment of A549 with HCI-2509 results in dysregulation of the cell cycle by regulating the expression of key regulators.

(A) Volcano plot of hu.Gene 2.0 Affymetrix micro array on untreated and HCI-2509-treated A549 cells. The cut-off was set to an ANOVA p-value of 0.05 and a fold-change of -1.5 < FC < 1.5. (B) 890 divergently expressed genes were found.

Supplemental Fig. S4

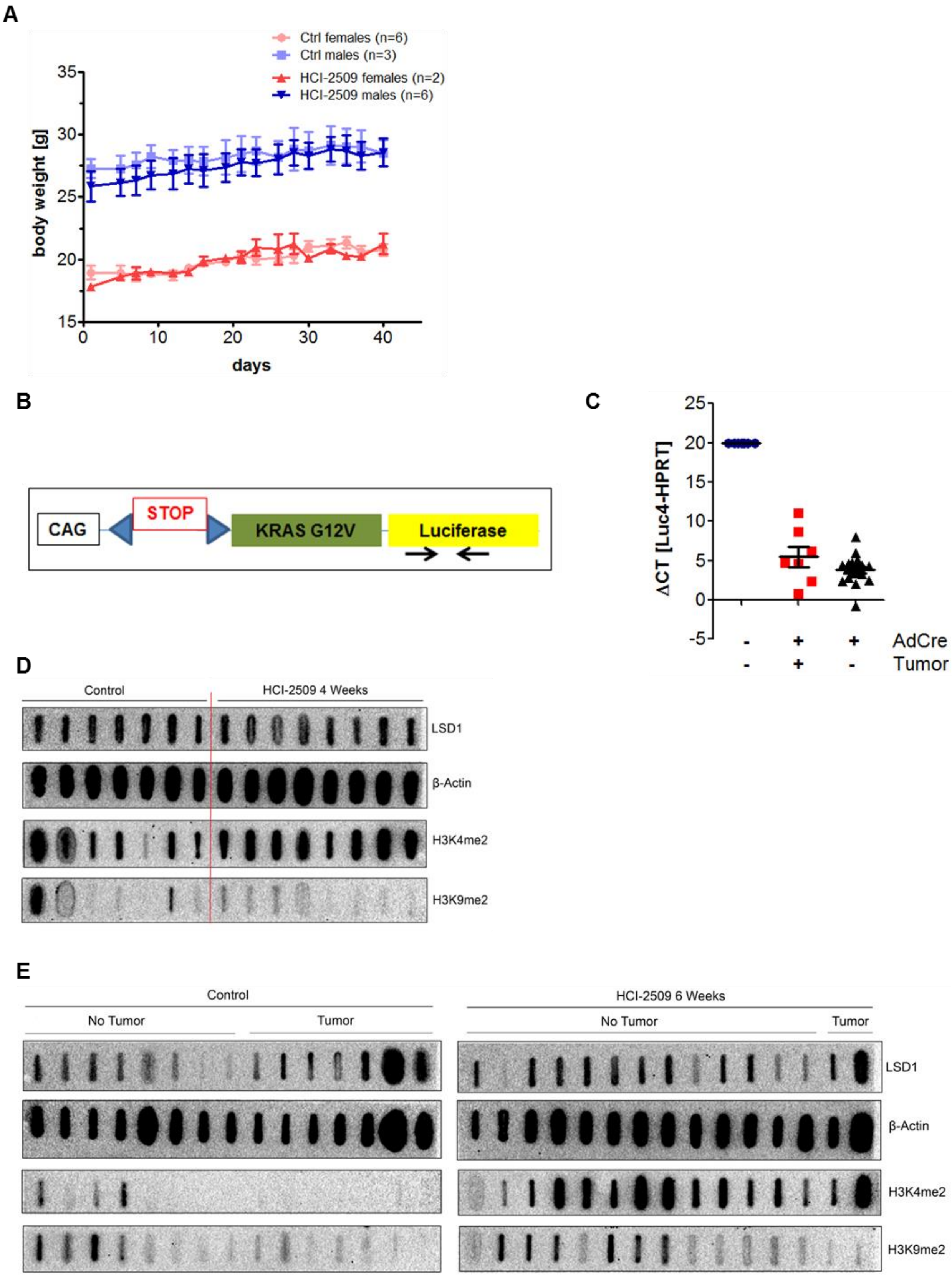

Supplemental Figure S4: Adverse side effects and adenoviral Cre application in C57BL/6N$^{(KRAS\ G12V)}$ mice was controlled.

(A) Mice treated and untreated (Ctrl) with HCI-2509 from both sexes were weighed every other day for 42 days. (B) After AdCre induction, the stop codon is excised and the mutated form of KRAS (KRAS G12V) and the luciferase reporter were expressed. DNAase-treated RNA from C57BL/6N$^{(KRAS\ G12V)}$ lungs were used for qPCR using primers that target the luciferase reporter (black arrows) RNA (see material and methods).

(C) The normalized expression of luciferase was used as a tumor induction control for all C57BL/6N$^{(KRAS\ G12V)}$ mice included in this study. Non-induced C57BL/6N$^{(KRAS\ G12V)}$ mice did not have an amplification for luciferase, and therefore, the Ct value for these mice was set to 40. ΔCt values for C57BL/6N$^{(KRAS\ G12V)}$ mice with visible tumors ranged between 1 and 15. A ΔCt value of 18 was set as a threshold and only C57BL/6N$^{(KRAS\ G12V)\ mice}$ with an ΔCt value of less than ΔCt 18 were included in this study.

(D) and (E) Square Blotting of protein lysate isolated from the lung of the EGFR (D) and KRAS (E) model treated either with control feed or feed containing HCI-2509, immunoblotted against LSD1, H3K4me2, H3K9me2, and the normalization protein ß-actin.
